# Capivasertib treatment associated with hypermetabolism of the spleen

**DOI:** 10.1186/s41824-024-00231-7

**Published:** 2025-03-24

**Authors:** Maria Ly, Gary Z. Yu, Amin Haghighat Jahromi

**Affiliations:** 1 Mallinckrodt Institute of Radiology, Washington University School of Medicine, St. Louis, Missouri, USA; 2 Radiology, Division of Nuclear Medicine, Mallinckrodt Institute of Radiology, Washington University School of Medicine in St. Louis, 510 South Kingshighway Blvd, Campus Box 8223, St Louis, Missouri, 63110 USA

**Keywords:** Capivasertib, Splenic hypermetabolism, Side effect, [18F]-FDG PET/CT

## Abstract

Capivasertib has emerged as a promising treatment for advanced breast and prostate cancer. Here, we report two cases of metastatic breast cancer patients who developed splenic hypermetabolism on [18 F]-FDG PET/CT following treatment with capivasertib. To our knowledge, these are the first documented instances of this treatment-related phenomenon. Although the mechanism behind this side effect remains unclear, these cases highlight an important imaging phenomenon that radiologists should consider when interpreting [18 F]-FDG PET/CT scans in patients treated with capivasertib.

Capivasertib is an oral selective pan-AKT inhibitor, which serves as a novel treatment option in advanced breast or prostate cancer in patients with PI3KCA, PTEN, or AKT1 alterations (Andrikopoulou et al. 2022; Luboff et al. 2024; Rugo et al. 2023). A phase III, randomized, double-blind trial demonstrated that treatment with the combination of capivasertib and fulvestrant in patients with hormone receptor+, HER-2-, AKT + breast cancer had significantly longer disease-free survival with a 50% reduced risk of disease progression or death when compared to Fulvestrant alone (Turner et al. 2023).

Here, we present two cases in which patients with metastatic breast cancer demonstrated splenic hypermetabolism on [^18^F]-Fluorodeoxyglucose ([^18^F]-FDG) PET/CT following treatment with Capivasertib. To our knowledge, these are the first documented instances of this treatment-related phenomenon.

The first patient is a 69-year-old woman with left breast invasive carcinoma (ER+, PR+, HER2-), status post bilateral mastectomy, adjuvant chest and axillary radiation, and aromatase inhibitor therapy, with two PIK3CA gain of function mutations. Given progression of disease and developing pneumonitis on fulvestrant and palbociclib, she was initiated on Capivasertib. [^18^F]-FDG PET/CT obtained three months later demonstrated interval development of hypermetabolic activity of the spleen without splenomegaly (Fig. 1A). Laboratory testing demonstrated no hematologic aberrancy or other explanation for splenic hypermetabolism.

**Fig. 1:**
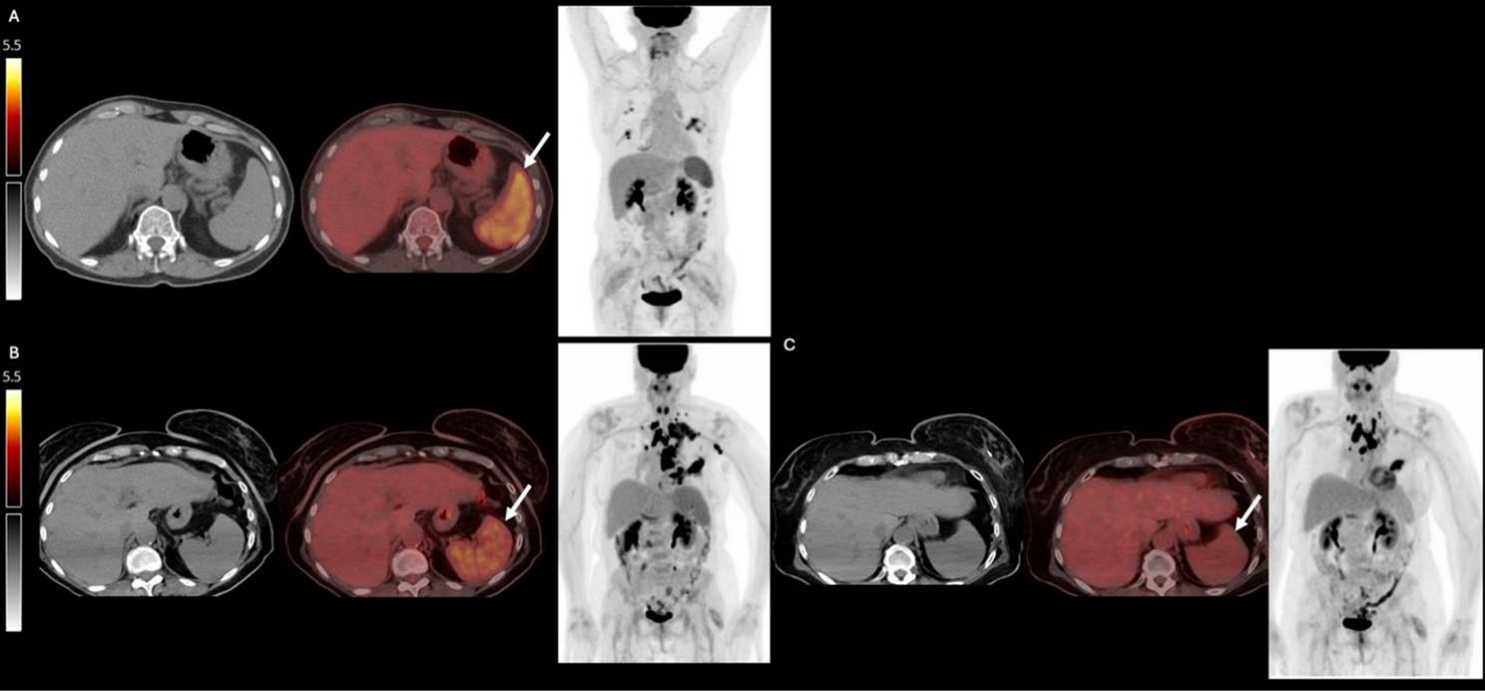
[^18^F] FDG-PET/CT axial CT, axial fused PET/CT, coronal PET maximum-intensity projection images are presented. (**A**) Patient 1 demonstrates hypermetabolic splenic activity following three months of capivasertib treatment. (**B**) Patient 2 demonstrates hypermetabolic splenic activity following three months of Capivasertib treatment. (**C**) Patient 2 demonstrates resolution of hypermetabolic splenic activity after change of regimen from capivasertib to capecitabine

The second patient is a 75-year-old woman with left breast invasive carcinoma (ER+, PR weak, HER2-, PIK3CA gain of function mutation, PTEN truncating mutation, two TP53 missense mutations), status post neoadjuvant docetaxel and cyclophosphamide, left breast conservation therapy, radiation therapy, and aromatase inhibitor therapy. Given progression of disease on fulvestrant and ribociclib, fulvestrant and capivasertib were initiated. [^18^F]-FDG PET/CT obtained three months later demonstrated progression of disease with enlarging hypermetabolic mediastinal, left axillary, supraclavicular nodes, pneumonitis, and also interval development of hypermetabolic activity of the spleen without splenomegaly (Fig. 1B). No hematologic aberrancy was noted. She was switched to capecitabine, with three month follow up [^18^F]-FDG PET/CT demonstrating resolution of the splenic hypermetabolic activity following cessation of capivasertib and favorable treatment response (Fig. 1C).

## Conclusion

To the best of our knowledge, these are the first documented cases of splenic hypermetabolism associated with capivasertib treatment. Although the exact mechanism underlying this phenomenon remains uncertain, radiologists should be vigilant when interpreting [18 F]-FDG-PET/CT scans in patients treated with capivasertib. Notably, neither of the patients was receiving immunotherapy, and neither exhibited bone marrow uptake, splenomegaly or hematologic abnormalities, suggesting that this phenomenon is unlikely to be related to systemic immune activation.

## Data Availability

Not applicable.
